# MR-guided ultra-hypofractionated radiotherapy for early-stage glottic cancer: A phase I feasibility study

**DOI:** 10.1016/j.ctro.2026.101297

**Published:** 2026-09-22

**Authors:** S.M.L. Linden, M.E.P. Philippens, J.A. Rijken, E. Brand, M.M.C. Schoenmakers, C.P.J. Raaijmakers, M.P.W. Intven, M. de Ridder

**Affiliations:** a Department of Radiotherapy, University Medical Center Utrecht, Utrecht, the Netherlands; b Department of Head and Neck Surgical Oncology, University Medical Center Utrecht, Utrecht, the Netherlands

**Keywords:** Ultra-hypofractionation, MR-guided radiotherapy, Early-stage glottic cancer, Motion management

## Abstract

**Introduction:**

Ultra-hypofractionation for early-stage glottic cancer reduces the number of hospital visits, but requires precise delivery to minimize the risk of radiation-induced toxicity. Magnetic Resonance (MR)-guided radiotherapy is a delivery technique that enables visualization of the tumor and laryngeal cartilages at each treatment session. In addition, motion management provides confidence that radiation is delivered precisely to the tumor while minimizing dose to the laryngeal cartilages. This study evaluates the feasibility and safety of an MR-guided ultra-hypofractionated treatment for early-stage glottic cancer.

**Study design:**

This is a prospective, single-center phase I feasibility study conducted at the University Medical Center (UMC) Utrecht. Twenty patients with cT1–2N0M0 squamous cell carcinoma of the glottic larynx and an indication for definitive radiotherapy will be enrolled. Active smokers and patients who do not meet the dose criteria will be excluded from the study. Patients receive 5 × 8.5 Gy, divided over three weeks (2 fractions per week). The GTV-PTV margin is 2 mm anterior-posterior, 2 mm left-right, and 4 mm cranio-caudal. The treatment is delivered on a 1.5 T MR-Linac (Elekta Unity), using online plan adaptation and motion management.

**Endpoints:**

The primary endpoints are technical feasibility and safety. MR-guided ultra-hypofractionated radiotherapy will be considered feasible if at least 80% of the patients (16/20 patients) successfully complete all five treatment fractions on the MR-Linac. The treatment is considered safe if a maximum of 10% of the patients (2/20 patients) experience a grade 3 disease-free late toxicity.

## Highlights

• Ultra-hypofractionation substantially reduces hospital visits.
• Patients receive 5 × 8.5 Gy over three weeks with two fractions weekly.
• Strict dose constraints are used for the laryngeal cartilages.
• Active smokers are excluded from the study.
• MR-guided radiotherapy enables laryngeal motion management during treatment.

## 1 Introduction

Glottic laryngeal cancer presents at an early stage (cT1–2) in about 80% of cases, due to voice changes [1]. Early-stage glottic cancer can be treated with either microscopic laser surgery or radiotherapy. Conventional radiotherapy treatment consists of 25–35 fractions, divided over 5–7 weeks, resulting in a recurrence-free survival of 86% [2] and low (severe) toxicity rates [3]. This long treatment schedule poses a considerable burden on patients and hospitals, emphasizing the need for more efficient treatment strategies.

Hospital visits can be significantly reduced when using an ultra-hypofractionated treatment schedule. However, reducing the number of fractions comes with a higher dose per fraction, potentially increasing the risk of radiation toxicity. Previous studies suggest that ultra-hypofractionated schedules for early-stage glottic cancer operate close to the threshold of acceptable toxicity, requiring a delicate balance between maximising tumor control and limiting normal-tissue damage [4], [5], [6], [7]. In a previous phase 1 dose-finding study conducted at the University of Texas (UT) Southwestern Medical Center (Dallas, Texas, United States of America) [8], both dose-limiting toxicities (2/29 patients) occurred in active smokers. This is in line with evidence that continued smoking during radiotherapy increases the risk of severe laryngeal toxicity [9]. Active smokers were therefore excluded from subsequent trials, and strict dose constraints were applied to the laryngeal cartilages. Subsequently, no severe late toxicity was observed in their phase II trial, where patients were treated with a regimen of 5 × 8.5 Gy, divided over three weeks [4]. These results suggest that, in non-smokers, this schedule stays on the favourable side of that delicate balance. Treatment was delivered using Cone Beam Computed Tomography (CBCT)-guided non-coplanar Volumetric Modulated Arc Therapy (VMAT). Surface guidance was used to account for laryngeal motion.

An alternative technique to deliver this ultra-hypofractionated treatment schedule is Magnetic Resonance (MR)-guided radiotherapy. Using MR-guided radiotherapy, the tumor and laryngeal cartilages are visualized each treatment, and radiotherapy plans are adapted accordingly. In addition, continuous MR imaging during treatment allows visualization of the larynx throughout radiation delivery. This enables laryngeal motion management and provides confidence that radiation is delivered to the tumor while minimizing dose to the laryngeal cartilages. The Adaptive Radiotherapy using Image guidance for Accurate treatment of glottic laryngeal cancer (ARIA) study evaluates the feasibility and safety of an MR-guided ultra-hypofractionated treatment for early-stage glottic cancer.

## 2 Study design

This is a prospective, single-center phase I feasibility study conducted at the University Medical Center (UMC) Utrecht. Twenty patients with cT1–2N0M0 squamous cell carcinoma of the glottic larynx and an indication for definitive radiotherapy will be enrolled. Patients who did not stop smoking at least two weeks prior to the start of treatment are excluded from the study. Eligibility criteria are summarized in Table 1.

**Table 1:** Inclusion and exclusion criteria.

Inclusion criteria	Exclusion criteria
Squamous cell carcinoma of the larynx.	Active smokers (did not stop at least 2 weeks prior to treatment).
cT1–2 stage.	Patients unsuited for MR imaging.
N0M0, based on bilateral ultrasound/MRI/PET.	Radiotherapy plan did not meet dosimetric constraints as defined in Table 2.
Indication for curative primary radiotherapy.	Patients receiving elective lymph node irradiation (ENI).
	Synchronous malignant tumor(s) at another site.
	Previous malignancies in the HN region treated with surgery (excluding microscopic laryngoscopy), chemotherapy, or radiotherapy. Patients who had a diagnostic or therapeutic MLS can be included in the study.
	Age < 18 years.
	WHO performance status >2.
	Participation in another interventional study.
	Mental or physical impairment causing the participant to be unable to fill out questionnaires. Or patients who are unwilling to fill out questionnaires.
	Concurrent chemotherapy or cetuximab.

### 2.1 Treatment description

#### 2.1.1 Simulation, imaging, delineation, and radiotherapy planning

All radiotherapy imaging will be done in a treatment mask, as in routine clinical practice. The pre-treatment MR and (pseudo-)CT scans will be used to delineate the Gross Tumor Volume (GTV) and organs at risk. In addition to the regular MR scan protocol, a high resolution T2 TSE mDixon MR-scan is acquired (Fig. 1, Supplementary file 2). The laryngeal cartilages (thyroid cartilage, cricoid cartilage, and arytenoid cartilages) are the main organs at risk. The Gross Tumor Volume (GTV)-Planning Target Volume (PTV) margin is 2 mm anterior-posterior, 2 mm left-right, and 4 mm cranio-caudal. No clinical target volume margin is used. Patients included in the study receive 5 × 8.5 Gy, divided over three weeks (two fractions per week). Strict dose constraints are used for the laryngeal cartilages (Table 2). These dose constraints are based on a previous phase II trial at UTSW Medical Center (Dallas, Texas) [4]. This study showed no severe toxicity and good oncological results for patients treated with a 5 × 8.5 Gy fractionation scheme.

**Fig. 1:**
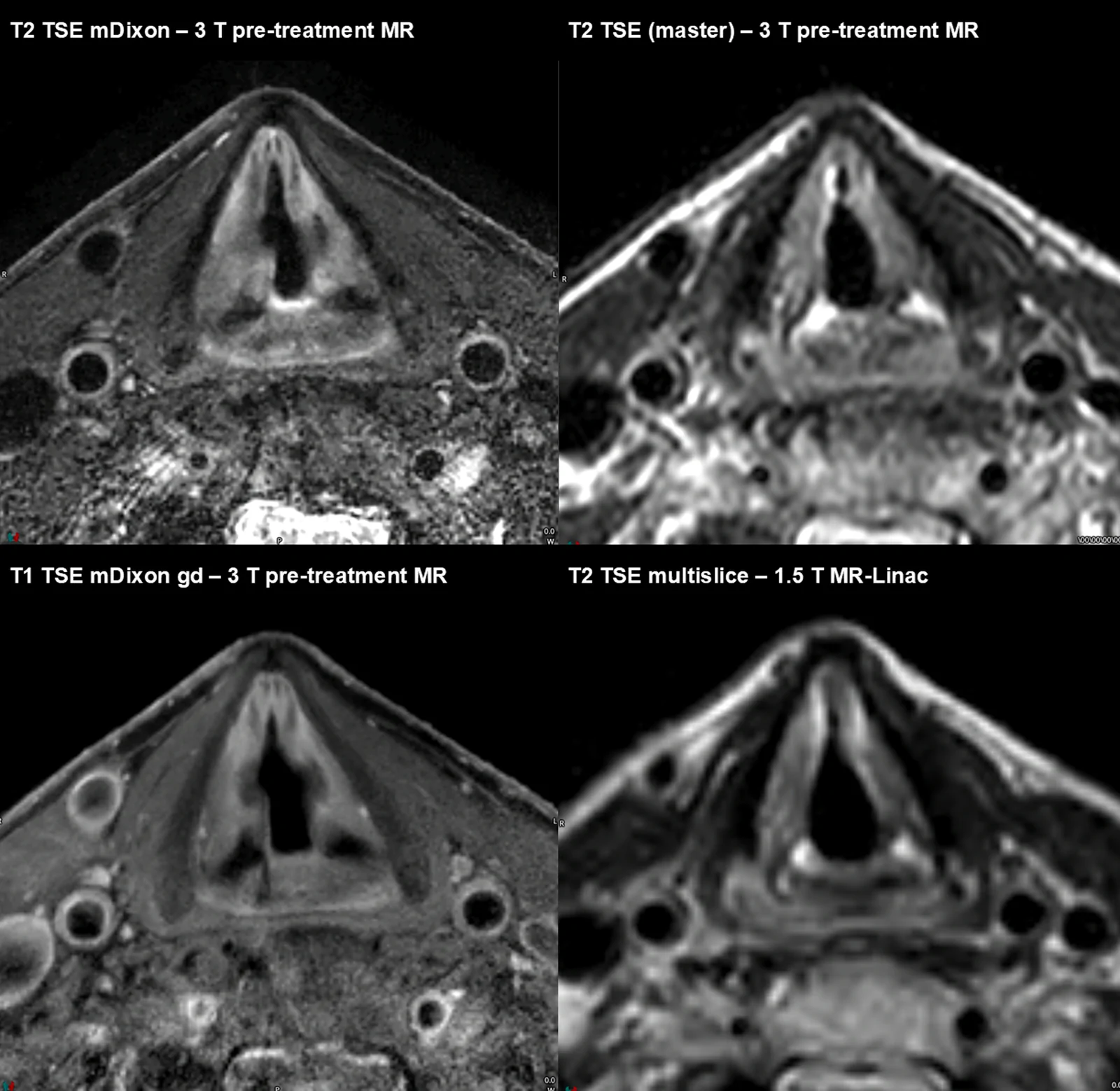
MR scans acquired on a 3 T MR scanner for pre-treatment delineation and MR scan acquired on the 1.5 T MR-Linac each fraction for daily plan adaptation.

**Table 2:** Dose constraints.

Structure	Constraint pre-treatment
GTV	V4250cGy ≥ 100%
PTV	V4250cGy ≥ 90% *Goal:* D0.03cc ≤ 49 Gy *Constraint:* D0.03cc ≤ 51 Gy
Ipsilateral arytenoid cartilage	Dmean ≤ 30 Gy D0.03cc ≤ 43 Gy
Contralateral arytenoid cartilage	*Goal:* Dmean ≤ 10 Gy D0.03cc ≤ 20 Gy *Constraint:* Dmean ≤ 30 Gy D0.03cc ≤ 43 Gy
Thyroid cartilage	D0.03cc ≤ 45.5 Gy
Cricoid cartilage	D0.03cc ≤ 45 Gy

#### 2.1.2 Daily treatment workflow

The treatment is delivered on a 1.5 T MR-Linac (Elekta Unity) (Fig. 2). After positioning in the treatment mask, a T2 TSE multislice scan is acquired (Fig. 1, Supplementary file 2). An adapt-to-shape (ATS) workflow is then performed, during which the target and organ-at-risk contours are reviewed and adapted to the anatomy of the day, followed by re-optimization of the treatment plan. After re-optimization, the dose to the laryngeal cartilages must not exceed the dose constraints by more than 5%.

**Fig. 2:**
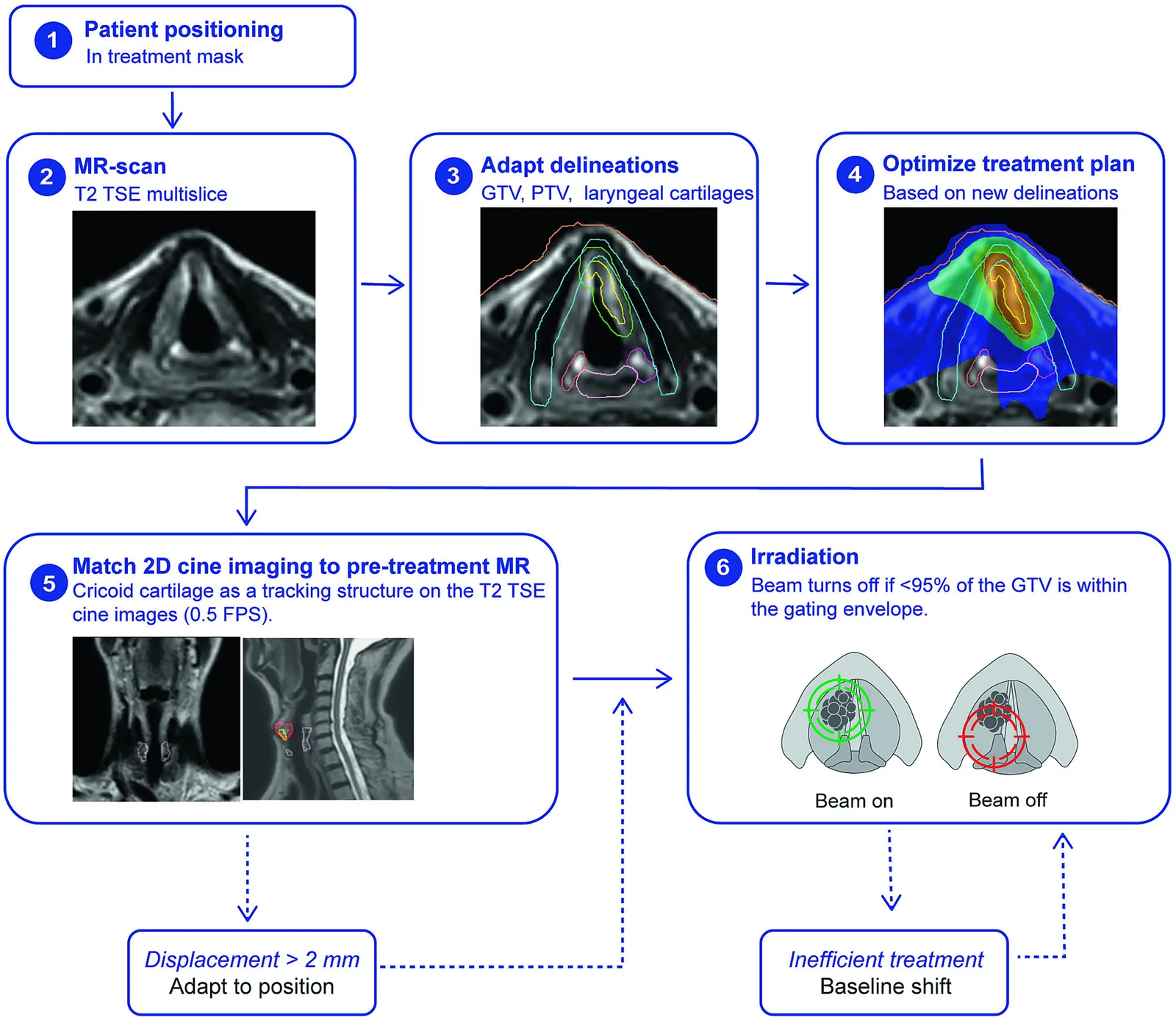
Online treatment workflow on the 1.5 T MR-Linac (Elekta Unity).

During radiation delivery, laryngeal motion is monitored using T2 TSE cine MRI (0.5 Frames Per Second), with the cricoid cartilage serving as a surrogate for the larynx. Immediately before irradiation, the position of the cricoid cartilage on cine MRI is matched to its position on the pretreatment scan. If displacement exceeds 2 mm, an adapt-to-position (ATP) procedure is performed to correct for this displacement. During irradiation, the beam is automatically interrupted whenever less than 95% of the GTV is contained within the gating envelope (Supplementary file 3). The gating envelope is a predefined region around the GTV within which the GTV must remain during radiation therapy. The gating envelope has a 3-mm margin to the lateral and posterior sides of the GTV and a 2-mm margin to the medial and anterior sides of the GTV. Cranio-caudal, a 4-mm margin is applied.

If a persistent displacement results in inefficient treatment delivery, a baseline shift is performed. The number of ATPs and baseline shifts per patient will be reported.

#### 2.1.3 Follow-up

Patients will be included in the regular follow-up with the radiation oncologist. They will be asked to fill out the Voice Handicap Index (VHI) questionnaire and quality of life questionnaires during the follow-up period. Late toxicity according to the Common Terminology Criteria for Adverse Events (CTCAE) v5 will be scored during regular follow-up.

### 2.2 Endpoints

#### 2.2.1 Primary endpoints

The primary endpoints are technical feasibility and safety. A treatment is considered technically feasible if all five fractions are completed on the MR-linac and each fraction meets all prespecified success criteria.

A fraction is considered successful if the following criteria are met:

• Total treatment time is < 1 hour. This threshold is based on prior experiences at other treatment sites within our institute [10].
• The dose on the laryngeal cartilages deviates less than 5% from the dose constraints (Table 2).
• The maximum dose on the PTV deviates less than 5% from the dose constraints and does not exceed a maximum of 120%.
• The patient completes the fraction without discontinuation because of treatment intolerance.

The treatment is considered safe if a maximum of 2/20 patients experience a grade 3 or higher treatment-related disease-free late toxicity. Treatment-related late toxicities include dysphagia, osteonecrosis, laryngeal edema, and pharyngeal necrosis. Radiation toxicity will be measured according to the CTCAE, which will be scored by the radiation oncologist at the start of RT and 3, 6, 12, and 24 months after treatment.

#### 2.2.2 Secondary endpoints

Secondary endpoints in this study are:

• Local recurrence occurring in the larynx at 2 years follow-up. Local recurrence is defined as histologically confirmed tumor recurrence at the site of the vocal cord where the primary tumor was located.
• Patient-reported outcomes regarding the tolerability of the treatment on the MR-Linac, measured by an MR-Linac patient-reported experience questionnaire at the first and last fraction [11].
• Voice quality, measured by the VHI questionnaire at the start of treatment, at 6 weeks, and 3, 6, 12, and 24 months after the treatment [12].
• Quality of life according to the European Organisation for Research and treatment of Cancer (EORTC) Quality of Life Questionnaire (QLQ-C30) and the EORTC Quality of Life Questionnaire for HNC cancer (QLQ-HN35). Patients will be invited to fill out questionnaires at baseline and 3, 6, 12, and 24 months after treatment.

### 2.3 Statistics

MR-guided ultra-hypofractionated radiotherapy will be considered feasible if at least 80% of the patients (16/20 patients) successfully complete all five treatment fractions on the MR-Linac. Treatment was considered infeasible for a patient if a single fraction was unsuccessful. Missed fractions will be replanned and the date of the replanned fraction will be saved. The treatment is considered safe if a maximum of 10% of the patients (2/20 patients) experience a grade 3 disease-free late toxicity at 2-year follow up. For the secondary endpoints, normally distributed continuous variables will be presented as mean with standard deviation. Non-normally distributed continuous variables will be presented as median with interquartile range.

### 2.4 Planned timeline

Radiation treatment of cT1–2 glottic laryngeal cancer is performed at the UMC Utrecht in approximately 20 cases/year. Based on previous studies conducted in our hospital for head and neck cancer patients, we estimate an accrual rate of 50%. Since we expect some delay due to patients who do not meet the criteria, a total of 2.5 years is estimated to complete treatment for 20 patients. Accrual started in November 2025, and is expected to be completed in May 2028. Completion of the 2-year follow-up therefore, is expected in May 2030.

## 3 Ethical and legal considerations

The study will be conducted according to the principles of the Declaration of Helsinki and in accordance with the Medical Research Involving Human Subjects Act (WMO) in the Netherlands. Each participant signs an informed consent form before the first study action. The Medical Ethics Trial Committee approved the study on 24-04-2025 (NL-009228).

Patients in the study will receive 5 fractions instead of 25 fractions, which is beneficial for patients. A single fraction of MR-guided radiotherapy will take longer compared to a conventional treatment fraction (30–60 min vs 15 min). If MR-guided ultra-hypofractionated radiotherapy is not feasible, a new radiation therapy plan will be made using the regular fractionation schedule on the conventional linac.

High doses on the OARs could result in increased radiation toxicity. The pre-treatment dose constraints are based on the study of Sher et al. [4], where radiation toxicity outcomes were promising (0% grade 3 or higher late toxicity). Additionally, we only include non-smoking patients, which is expected to be one of the largest risk factors for developing radiation toxicity. The informed consent form includes a specific statement confirming that participants understand that active smokers are not eligible for participation. We expect that we have minimized the risks of high-grade radiation toxicity.

## Registration details

Dutch registry of clinical trials (CCMO): NL88385.041.25. NedMec protocol number: NL-009228 (approved on 24-04-2025).

## CRediT authorship contribution statement

**S.M.L. Linden:** Writing – original draft, Visualization, Supervision, Project administration, Methodology, Conceptualization. **M.E.P. Philippens:** Writing – review & editing, Visualization, Supervision, Methodology, Conceptualization. **J.A. Rijken:** Writing – review & editing, Conceptualization. **E. Brand:** Writing – review & editing, Methodology, Conceptualization. **M.M.C. Schoenmakers:** Writing – review & editing, Methodology, Conceptualization. **C.P.J. Raaijmakers:** Writing – review & editing, Visualization, Supervision, Methodology, Conceptualization. **M.P.W. Intven:** Writing – review & editing, Visualization, Supervision, Methodology, Conceptualization. **M. de Ridder:** Writing – review & editing, Visualization, Supervision, Project administration, Methodology, Funding acquisition, Conceptualization.

## Funding

This research was funded by 10.13039/501100003761UMC Utrecht.

## Declaration of competing interest

Our department has a research agreement with Elekta AB. Elekta AB had no role in the preparation, review, or approval of the manuscript.
