# Supplementary material for: MR-guided ultra-hypofractionated radiotherapy for early-stage glottic cancer: A phase I feasibility study
Source: Clin Transl Radiat Oncol. 2026 Sep 22;62:101297. doi: 10.1016/j.ctro.2026.101297 (PMC13635521; doi:10.1016/j.ctro.2026.101297)
Supplement: Supplementary file 1 [file mmc1.pdf]

Adaptive Radiotherapy using Image guidance for Accurate treatment of glottic laryngeal cancer (ARIA)

A feasibility study

Version 1.2

(January 2026)

Protocol title: Adaptive Radiotherapy using Image guidance for Accurate treatment of glottic laryngeal cancer (ARIA)

**Protocol ID**	**NL-009228**
**Short title**	**ARIA**
**Version**	**1.2**
**Date**	**23-01-2026**
**Principal investigator(s)**	***Mischa de Ridder, MD, PhD, Radiation Oncologist***
**Sponsor (in Dutch: verrichter/opdrachtgever)**	***University Medical Centre Utrecht***<br>***Department of Radiotherapy***<br>***Heidelberglaan 100***<br>***3584 CX Utrecht, The Netherlands***
Subsidising party	***UMC Utrecht***
**Independent expert (s)**	***Judith Roesink, MD, PhD, Radiation Oncologist***

PROTOCOL SIGNATURE SHEET

Name	Signature	Date
**Manager Research**<br>*Prof. Dr. D.W.J. Klomp*		**23-01-2026**
Principal investigator:<br>*Mischa de Ridder, MD, PhD, Radiation Oncologist*		**23-01-2026**

TABLE OF CONTENTS

1. INTRODUCTION AND RATIONALE 9
1.1 Problem description 9
1.2 Aim/hypothesis 9
1.3 Literature 9
2. OBJECTIVES 12
2.1 Primary Objective 12
2.2 Secundary Objective(s) 12
3. STUDY DESIGN 13
3.1 Study design 13
3.2 Duration of the study 13
4. STUDY POPULATION 14
4.1 Population (base) 14
4.2 Inclusion criteria 14
4.3 Exclusion criteria 14
4.4 Sample size calculation 14
5. TREATMENT OF RESEARCH PARTICIPANTS 15
5.1 Investigational treatment 15
6. METHODS 16
6.1 Study parameters/endpoints 16
6.1.1. Main study parameter/endpoint 16
6.1.2 Secondary study parameters/endpoints 16
6.1.3 Other study parameters 16
6.2 Study procedures 17
6.2.1 Main procedures 17
6.2.2 Measurements 19
6.2 Withdrawal of individual research participants 19
6.3.1 Specific criteria for withdrawal 19
6.3 Replacement of individual subjects after withdrawal 20
6.4 Follow-up of subjects withdrawn from treatment 20
6.5 Premature termination of the study 20
7. SAFETY REPORTING 21
7.1 Temporary halt for reasons of research participants safety 21
7.2 AEs, SAEs and SUSARs 21
7.2.1 Adverse events (AEs) 21
7.2.2 Serious adverse events (SAEs) 21
7.3 Follow-up of adverse events 22
7.4 Safety Committee/DSMB 22
8. STATISTICAL ANALYSIS 23
8.1 Primary study parameter(s) 23
8.3 Other study parameters 23
9. ETHICAL CONSIDERATIONS 24

9.1 Regulation statement 24
9.2 Recruitment and consent 24
9.3 Benefits and risks assessment, group relatedness 24
9.4 Compensation for injury 25
9.5 Incentives and reimbursements 25
10. ADMINISTRATIVE ASPECTS, MONITORING AND PUBLICATION 26
10.1 Handling and storage of data and documents 26
10.2 Monitoring and Quality Assurance 27
11. STRUCTURED RISK ANALYSIS 30
12. REFERENCES 31

LIST OF ABBREVIATIONS AND RELEVANT DEFINITIONS

**ABR**	**General Assessment and Registration form (ABR form), the application form that is required for submission to the accredited Ethics Committee; in Dutch: Algemeen Beoordelings- en Registratieformulier (ABR-formulier)**
**AE**	**Adverse Event**
**AR**	**Adverse Reaction**
**AP**	**Anterior-Posterior**
**ATS**	**Adapt To Shape**
**CA**	**Competent Authority**
**CCMO**	**Central Committee on Research Involving Human Subjects; in Dutch: Centrale Commissie Mensgebonden Onderzoek**
**CV**	**Curriculum Vitae**
**CC**	**Cranial-Caudal**
**CMM**	**Comprehensive Motion Manegement**
**DSMB**	**Data Safety Monitoring Board**
**EU**	**European Union**
**EudraCT**	**European drug regulatory affairs Clinical Trials**
**GCP**	**Good Clinical Practice**
**GDPR**	**General Data Protection Regulation; in Dutch: Algemene Verordening Gegevensbescherming (AVG)**
**GTV**	**Gross Tumour Volume**
**IB**	**Investigator’s Brochure**
**IC**	**Informed Consent**
**IMP**	**Investigational Medicinal Product**
**IMPD**	**Investigational Medicinal Product Dossier**
**LR**	**Left-Right**
**METC**	**Medical research ethics committee (MREC); in Dutch: medisch-ethische toetsingscommissie (METC)**
**MRI**	**Magnetic Resonance Imaging**
**OARs**	**Organs At Risk**
**PTV**	**Planning Target Volume**
**PROs**	**Patient Reported Outcomes**
**SABR**	**Stereotactic Ablative Body Radiotherapy**
**(S)AE**	**(Serious) Adverse Event**

**SPC**	**Summary of Product Characteristics; in Dutch: officiële productinformatie IB1-tekst**
**Sponsor**	**The sponsor is the party that commissions the organisation or performance of the research, for example a pharmaceutical company, academic hospital, scientific organisation or investigator. A party that provides funding for a study but does not commission it is not regarded as the sponsor, but referred to as a subsidising party.**
**SUSAR**	**Suspected Unexpected Serious Adverse Reaction**
**UAVG**	**Dutch Act on Implementation of the General Data Protection Regulation; in Dutch: Uitvoeringswet AVG**
**WMO**	**Medical Research Involving Human Subjects Act; in Dutch: Wet Medisch-wetenschappelijk Onderzoek met Mensen**

SUMMARY

Rationale: In order to decrease the number of radiotherapy fractions in cT1-2 stage glottic larynx cancer patients, a stereotactic approach on magnetic resonance guided linear accelerator (MR-Linac) is proposed. This treatment approach consists of 5 radiation fractions (8.5 Gy/fx) instead of the conventional 25 fractions (2.4 Gy/fx).

Objective: *Primary objective:* study technical feasibility and safety to perform stereotactic radiotherapy on MR-linac for cT1-2 glottic cancer patients. *Secondary objectives:* monitor patient safety, toxicity and tolerability of this treatment concept.

Study design: Phase I feasibility study

Study population: Non-smoking patients with cT1-2 glottic larynx cancer who are eligible for treatment with a fractionation scheme of 25x2.4 Gy are eligible for inclusion.

Intervention: Patients who are able to meet the pre-treatment dose constraints will be treated with 5 fractions divided in 2 weeks. The primary tumour will be treated with 5x8.5 Gy (42.5 Gy). Doses on the laryngeal cartilages will be kept as low as reasonably possible. All treatment fractions will be delivered on the MR-linac, which is a linear accelerator combined with an MRI (magnetic resonance imaging). The MR-linac facilitates the integration of MRI in the radiotherapy planning and treatment. This enables position and shape adaptation for the treatment plan of each fraction and motion management during each fraction.

Main study parameters/endpoints: The percentage of treatment fractions successfully delivered on MR-Linac.

Nature and extent of the burden and risks associated with participation, benefit and group relatedness:

Benefits: less treatment fractions (5 vs 25) with potentially an improved oncological outcome.

Burdensome: treatment time on the MR-linac will be longer (max 60 min. vs 15 min per fraction).

Risks: This is a moderate risk study. Higher doses on the larynx may potentially lead to a higher toxicity outcome. However, since we only include non-smoking patients that meet the pre-defined dose constraints, we expect to have reduced this risk.

1. INTRODUCTION AND RATIONALE

1.1 Problem description

Glottic laryngeal cancer, with an annual incidence of 400-450 cases in the Netherlands, often presents at an early stage (cT1-2 N0) due to voice changes caused by minor irregularities in the vocal cords. At UMC Utrecht, around 30-40 patients are diagnosed with early-stage glottic cancer each year. The prognosis for these patients is generally excellent, with a disease-specific survival rate of 90-95% at 5 years. (1) Given this good prognosis, minimizing treatment-related side effects is crucial.

Currently, two primary treatment options exist for this disease: transoral microscopic laser surgery (MLS) and radiotherapy. Radiotherapy is thought to better preserve voice quality in more extensive early-stage glottic cancers, where for patients with limited T1a tumours MLS is often preferred. However, radiotherapy treatment requires patients to undergo a regimen of at least 25 treatment fractions over 5 weeks up to 35 fractions over 7 weeks. This poses a considerable burden on patients and hospitals, leading to the need for optimized treatment strategies to reduce this treatment time.

Stereotactic radiotherapy lowers the number of radiotherapy fractions, by treating with higher doses per fraction. Higher doses per fraction could cause more radiation toxicity, which is why the dose on the surrounding structures should be minimized. In order to maximize this dose reduction, MR-linac will be used enable treatment plan adaptation for each fraction, monitor motion during the treatment and thereby minimize the PTV (Planning Target Volume) margins, that account for motion during treatment and setup uncertainty.

1.2 Aim/hypothesis

The aim of this study will be to examine if stereotactic radiotherapy for early-stage glottic laryngeal cancer is feasible using MR-Linac. The hypothesis of the study is that stereotactic radiotherapy of early stage glottic cancer is feasible on MR-Linac

1.3 Literature

A phase II study from the group of UT Southwestern shows promising results of stereotactic ablative body radiotherapy (SABR) scheme, using non-coplanar CBCT-guided Linacs for T1-2 glottic larynx cancer. (2) In this study, patients who do not smoke actively and with a PTV < 10 cc were treated with 42.5 Gy in 5 fractions, twice per week. Other patients received 58.08 Gy in 16 daily fractions (3.63 Gy/fraction), which was also investigated in a study performed by Erasmus MC Cancer Institute. (2) Both studies lowered the dose on the whole larynx and

irradiated the Gross Tumour Volume (GTV) with a high dose. Lowering the dose on the whole larynx results in less radiation toxicity. Grade 3 disease-free late radiation toxicity occurred in 0% (0/25) and 6.5 % (7/111) of the patients, respectively for these studies. In both studies, no Grade 4 or 5 late toxicity occurred. In current practice at UMC Utrecht, grade 3 or higher late toxicity is 4.3% (1) .

Another phase I dose escalating study of SABR was terminated due to dose-limiting toxicities. (3) The study reported 2 of 6 patients with grade 3 late toxicity, receiving 55 Gy in 11 fractions. This study irradiated the whole larynx to much higher doses in comparison to the SABR study performed at UT Southwestern. A comparison of the outcomes and prescribed doses is presented in table 1.

Table 1 Hypofractionated radiation toxicities and dose evaluation in literature.
*(*Alpa/beta=3 is used to calculate the EQD2 on the organs at risk)*

**Prescribed dose**	**42.5 Gy / 5 fx**	**58.08 Gy /16 fx**	**58.08 Gy /16 fx**	**59.5 Gy /17 fx**	**55 Gy /11 fx**	**Planning study MR-Linac 42.5 Gy/5 fx**
Study	Sher et al. (2)	Sher et al. (2)	Tans et al. (3)	Kang et al. (4)	Kang et al. (4)	
Number of patients (n=)	21	4	111	7	6	5
Prescribed PTV dose (EQD2, α/β=10 Gy)	65.52 Gy	65.97 Gy	65.97 Gy	66.94 Gy	68.75	65.52
Ipsilateral arytenoid (Dmean)*	32.60 Gy (median)	55.99 Gy (median)	*Not described*	77.4 Gy (mean)	90.1 Gy (mean)	44.67 Gy (median)
Contralateral arytenoid (Dmean)*	12.04 Gy (median)	17.55 Gy (median)	26.60 Gy (median)	67.3 Gy (mean)	71 Gy (mean)	16.22 Gy (median)
Thyroid cartilage *	Dmax = 107.56 Gy (median)	Dmax = 84.39 Gy (median)	*Not described*	Dmean = 83.46 Gy (mean)	Dmean = 129.21 Gy (mean)	Dmax = 106.74 Gy (median)
Cricoid cartilage *	Dmax = 96.55 Gy (median)	Dmax = 82.26 Gy (median)	*Not described*	Dmean = 56.6 Gy (mean)	Dmean = 108.81 Gy (mean)	Dmax = 78.21 Gy (median)
**Median Follow-up**	3.7 years (3.1-4.4)	3.7 years (3.1-4.4)	41 months (8-84)	17.1 months (4.8-21.5)	17.1 months (4.8-21.5)	-
**Local Recurrences**	1/21	1/4	2/111	0/7	1/6	-
**Late Grade 3 toxicity**	0/21	0/4	7/111	0/6	2/6	-

For the present study, the dose constraints will be based on the phase II trial of Sher et al. for radiotherapy planning. Patients will be excluded if they cannot meet the dosimetric constraints as provided in table 2.

Tabel 2 Dosimetric constraints. If a patient cannot meet these criteria pre-treament, the patient will not be included in the study.

Structure	Constraint pre-treatment
GTV	V4250cGy >= 100%
PTV	V4250cGy >= 90%<br>Dmax 0.03cc <= 5100 cGy
Arytenoid cartilages	Dmax 0.03cc <= 4300 cGy<br>Dmean <= 3000 cGy
Thyroid cartilage	Dmax 0.03cc <= 4550 cGy
Cricoid cartilage	Dmax 0.03 cc<= 4500 cGy

2. OBJECTIVES

2.1 Primary Objective

Study the technical feasibility and grade 3 late radiation toxicity (according to CTCAE) of stereotactic radiation therapy for cT1-2 glottic laryngeal cancer on the MR-Linac. Stereotactic radiation therapy is defined as 5 fractions of 8.5 Gy.

2.2 Secundary Objective(s)

Monitor stereotactic radiation therapy for early-stage glottic cancer regarding:

- Patient safety: local control
- Voice quality (Voice Handicap Index)
- Patient reported outcomes (PROs)
  - Treatment tolerability questionnaires (MRL-PRE)
  - Quality of life questionnaires

3. STUDY DESIGN

3.1 Study design

This is a monocenter phase I feasibility study, that will be performed at UMC Utrecht. Twenty patients with cT1-2N0M0 glottic laryngeal carcinoma will be treated according the new treatment design, using stereotactic radiation therapy.

3.2 Duration of the study

Radiation treatment of cT1-2 glottic laryngeal cancer is performed in the UMC Utrecht in approximately 20 cases each year. Based on previous studies conducted in our hospital for head and neck cancer patients, we estimate an accrual rate of 50%. Since we expect some delay due to exclusions of active smokers and patients who do not meet predefined dose criteria, a total of 2.5 years will be necessary to complete treatment for 20 patients. With a follow-up of at least 2 years, the study will last 4.5 years

4. STUDY POPULATION

4.1 Population (base)

Patients with cT1-2 N0M0 glottic laryngeal cancer.

4.2 Inclusion criteria

In order to be eligible to participate in this study, a subject must meet all of the following criteria:

- Squamous cell carcinoma of the larynx
- cT1-2 stage
- N0, M0, based on bilateral ultrasound / MRI / PET of the neck
- Indication for curative primary RT

4.3 Exclusion criteria

A potential subject who meets any of the following criteria will be excluded from participation in this study:

- Concurrent chemotherapy or cetuximab
- Did not stop smoking at least 2 weeks before start of treatment (patients who have never smoked will be eligible for inclusion)
- Patients unsuited for MRI imaging
- Synchronous malignant tumour(s) at another site
- Previous malignancies in the HN region treated with surgery (excluding microscopic laryngoscopy (MLS)), chemotherapy, or radiotherapy. Patients who had a diagnostic or therapeutic MLS can be included in the study.
- Age < 18 years
- WHO performance status > 2
- Participation in another interventional study
- Did not meet primary dosimetric constraints as defined in section 1 (table 2)
- Mental or physical impairment causing the participant to be unable to fill out questionnaires
- Patients who are not willing to fill in questionnaires

4.4 Sample size calculation

Since this is a feasibility study no sample size was calculated. Based on our experience with phase I studies we estimate a sample size of 20 patients will be sufficient to achieve the study objectives.

5. TREATMENT OF RESEARCH PARTICIPANTS

5.1 Investigational treatment

Delineation of target areas

In routine practice, all radiotherapy imaging is done in a treatment mask. For this study, a double shell positioning system (DSPS) mask will be used for all patients. The pre-treatment CT and/or MR-scans will be used to delineate the tumour and Organs At Risk (OARs), including the cartilages of the larynx. The radiation oncologist will review all delineations and make adjustment if necessary.

Position verification and margins used for the primary tumour

As in common clinical practice, no CTV margin will be added for Stereotactic Ablative Radiotherapy (SABR) treatments. A margin of 2 mm to the GTV in anterior-posterior (AP) and Left-right (LR) direction will be used for the to create a PTV. In de Cranial-Caudal (CC) direction, the margin to the GTV will be 3-5 mm, depending on the movement of the larynx during the respiratory cycle. The PTV will be treated with 5x8.5 Gy using the dose constraints as described in table 2, according to the Phase II clinical trial of Sher et al. (4) All fractions will be applied on the MR-Linac, which is a linear accelerator combined with an MRI (Magnetic Resonance Imaging).

Treatment adaptation on the MR-Linac

Using the MR-linac it is possible to adapt the treatment plan while the patient is on the treatment table. The adapt to shape (ATS) workflow allows for adaptation of all contour of target areas and OARs based on the new patient anatomy as seen on MRI imaging performed on the MR-linac. An adapted RT plan is created while the patient is on the treatment table. Since the treatment consists of only 5 fractions, the ATS procedure will be performed online for each fraction to enable accurate shape adaptation.

Intrafraction motion on the MR-Linac

The feasibility of Comprehensive Motion Management (CMM) will be investigated to enable real-time tracking of the larynx and automatic gating during treatment. This approach has the potential to enhance treatment precision by accounting for tumour movement during treatment. If CMM seems feasible for the larynx, this will be used.

6. METHODS

6.1 Study parameters/endpoints

6.1.1. Main study parameter/endpoint

The percentage of patients treated successfully with 5 fractions performed on the MR-linac. A fraction is technical feasible if:

- Treatment time is <1 hour. The (ATS) workflow includes: MR-scans for position verification, evaluation and adaptation of the contours by a radiation oncologist, re-optimizing the treatment plan and radiation dose delivery.
- The doses on the OARs deviates less than 5% from the pre-treatment plan doses.
- The maximum dose on the PTV deviates less than 5% from the pre-treatment plan dose and does not exceed a maximum of 120%.
- Patients can endure the treatment

The treatment is safe if a maximum of 2/20 patients experience a grade 3 disease-free late toxicity. Radiation toxicity will be measured according to the Common Terminology Criteria for Adverse Events (CTCAE) which will be filled in by the radiation oncologist at the start of RT and 3, 6, 12 and 24 months after treatment.

6.1.2 Secondary study parameters/endpoints

Regarding patient safety:

- Local recurrence occurring in the larynx at 2 years follow-up

Regarding patient tolerability for MR-Linac and quality of life:

- Patient reported outcomes (PROs) regarding tolerability of treatment on MR-linac will be measured with the validated MRL patient reported experience questionnaire of two time points: after the first and the last fraction
- Quality of life scoring: EORTC Quality of Life Questionnaire (QLQ-C30) and the EORTC Quality of Life Questionnaire HNC cancer (QLQ-HN35). Patients will be invited to fill out questionnaires at baseline and 3, 6, 12 and 24 months after treatment.

Regarding voice quality:

- Voice Handicap Index (VHI) will be filled in before the start of treatment, at 6 weeks, 3, 6, 12 and 24 months after the treatment.

6.1.3 Other study parameters

- Patient and tumour characteristics
- All images obtained before and during treatment

- Treatment characteristics: dosimetric and planning data, treatment duration per fraction, percentage of fractions delivered as planned, visibility and change of contours of the tumour on MRI.

6.2 Study procedures

6.2.1 Main procedures

1. *Selection of patients:* Eligible patients with cT1-2 glottic larynx carcinoma will be approached by their radiation oncologist and ask the patient if a researcher may contact them for study information.. Patients that are interested will be informed by the responsible investigator about the study procedures and possible risks and benefits and reach out the study information letter. Patients willing to participate sign an informed consent form together with the responsible researcher.
2. *Preparation and treatment according to the conventional treatment:* For all HNC patients undergoing conventional treatment, a personal fixation mask is made. Thereafter, patients will undergo Computed Tomography (CT) and MRI in the fixation mask. The radiation oncologist will delineate the target areas and OARs.
3. *Additional preparation and treatment according to the new concept:* Additional to the conventional OARs, the laryngeal cartilages (arytenoid cartilage, thyroid cartilage and cricoid cartilage) will be delineated on CT or MRI as additional organs at risk. The MRI and CT will be used to create a stereotactic RT plan containing of 5 fractions in 2 weeks. A back-up plan with conventional 25 fraction will be created for if a fraction on MRL is not technical feasible. In that case the patient will continue treatment using the standard treatment plan (25x2.4 Gy). MRL questionnaires about the treatment on the MR-Linac will be obtained after the 1st and 5th fraction (MRL-PRE). This questionnaire is previously validated and previously used for investigating patient experience on de MR-Linac. (5, 6) At each fraction, the patient will be asked if they smoked since the last fraction.
4. Follow-up procedures*:* patients will be included in the regular follow-up program. During the regular follow-up, the VHI questionnaire will be filled in together with the radiation oncologist. The quality of life questionnaires will be send to the patients, according to the MOMENTUM study (METC number: 18-620). Patients who are not willing to fill in questionnaires will be excluded from the study.

Study procedures ARIA study

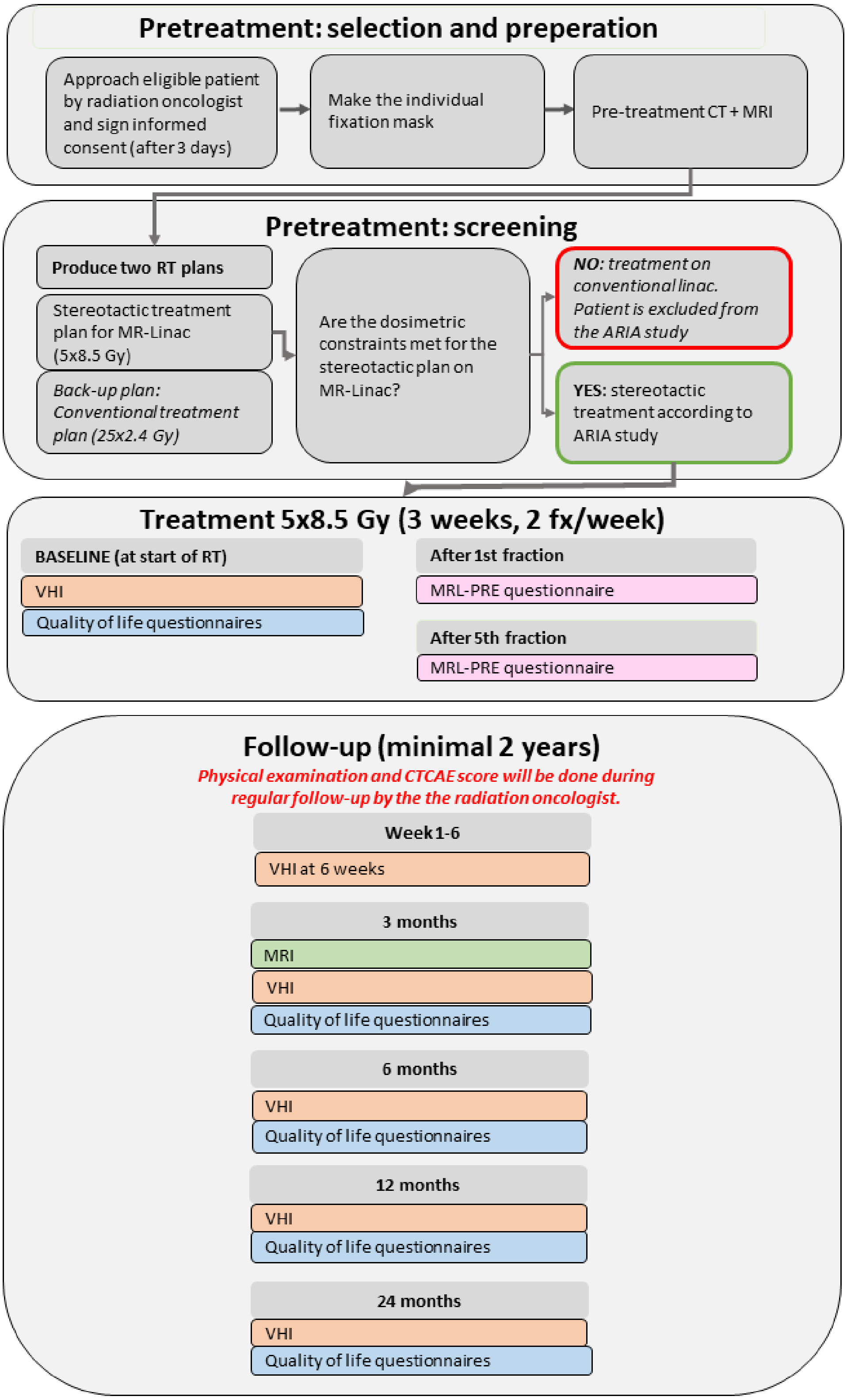

6.2.2 Measurements

Before treatment:

Patients willing to participate will sign an informed consent form together with the responsible researcher. After signing IC, patients will fill in the Voice Handicap Index (VHI), consisting of 30 questions about their voice and the effect of their voice on their life. It will take approximately 5 minutes to fill out the questionnaire at each time point. The quality of life questionnaires will be send to the patients before treatment (more information about these questionnaires in *After treatment*)

During treatment

Specific MRL patient reported experience (MRL-PRE) questionnaires will be used to assess the patient experience during MR-linac treatment. The MRL-PRE questionnaires has been used in a previous study in the UMC-Utrecht that was approved by the METC Utrecht (PRECIEVE, METC-protocol number: 20-624/C). Questionnaires will be obtained after the first and the last fraction on the MR-linac. It will take approximately 10 minutes to fill out the questionnaires at each time point.

After treatment

Patients will be invited to fill out quality of life questionnaires at baseline and 3, 6, 12 and 24 months after radiotherapy. Two questionnaires will be obtained: the EORTC Quality of Life Questionnaire (QLQ-C30) and the EORTC Quality of Life Questionnaire HNC cancer (QLQ-HN35). The QLQ-C30 and the QLQ-HN35 are used in previous approved studies such as the MOMENTUM study (METC number: 18-620). Answering all questionnaires will take approximately 20 minutes at each time point.

6.2 <u>Withdrawal of individual research participants</u>

Participants can leave the study at any time for any reason without any consequences if they wish to do so. The investigator can decide to withdraw a participant from the study for urgent medical reasons. Patients who are included in the study and decide to withdraw before start of treatment will be replaced by another participant. Patients who withdraw during treatment will be evaluated.

6.3.1 Specific criteria for withdrawal

Patients that smoke during treatment are withdrawn from the study. These patients will be excluded for further analysis.

6.3 Replacement of individual subjects after withdrawal

Patients that withdraw will be replaced unless patients stop since they cannot endure treatment.

6.4 Follow-up of subjects withdrawn from treatment

Subjects withdrawn from treatment will be included in the routine oncological follow-up.

6.5 Premature termination of the study

In case the study ended prematurely, the investigator will notify the accredited METC, including the reasons for the premature termination.
The study will be stopped if:

1. After 10 inclusions, 2 patients (10%) encounter grade 3 or higher late laryngeal radiation toxicity (>3 months), according to the CTCAE v.5.
2. More than 2 out of 20 patients (>10%) are not able to finish 5 treatment fractions on the MR-linac.
3. More than 2 out of 20 patients (>10%) will have a local recurrence in the larynx within two years after treatment.
4. More than 2 out of 20 patients (>10%) encounter grade 3 or higher late laryngeal radiation toxicity (>3 months), according to the CTCAE v.5.

The stopping criteria will be checked every 6 months to ensure the safety and tolerability of this trial.

7. SAFETY REPORTING

7.1 Temporary halt for reasons of research participants safety

In accordance to section 10, subsection 4, of the WMO, the sponsor will suspend the study if there is sufficient ground that continuation of the study will jeopardise subject health or safety. The sponsor will notify the accredited METC without undue delay of a temporary halt including the reason for such an action. The study will be suspended pending a further positive decision by the accredited METC. The investigator will take care that all subjects are kept informed.

7.2 AEs, SAEs and SUSARs

7.2.1 Adverse events (AEs)

Adverse events are defined as any undesirable experience occurring to a subject during the study, whether or not considered related to the experimental intervention.
All patients that are treated with radiotherapy are likely to experience several known acute and long term complications, which could be marked as adverse events. Examples of acute RT are: mucositis, dysphagia, xerostomia, dermatitis, fatigue, weight loss and mild/moderate fever. Examples of long term RT related toxicities in HNC patients are: xerostomia, dysphagia, hypothyroidism, carotid stenosis, skin fibrosis and fatigue. These known complications will not be reported as adverse events in this study. Any other adverse events reported spontaneously by the subject or observed by the investigator or his staff will be recorded.

7.2.2 Serious adverse events (SAEs)

A serious adverse event is any untoward medical occurrence or effect that

- results in death;
- is life threatening (at the time of the event);
- requires hospitalisation or prolongation of existing inpatients' hospitalisation;
- results in persistent or significant disability or incapacity;
- is a congenital anomaly or birth defect; or
- any other important medical event that did not result in any of the outcomes listed above due to medical or surgical intervention but could have been based upon appropriate judgement by the investigator.

An elective hospital admission will not be considered as a serious adverse event.

The sponsor will report the SAEs through the web portal onderzoeksportaal to the accredited METC that approved the protocol, within 7 days of first knowledge for SAEs that result in

death or are life threatening followed by a period of maximum of 8 days to complete the initial preliminary report. All other SAEs will be reported within a period of maximum 15 days after the sponsor has first knowledge of the serious adverse events.

7.3 Follow-up of adverse events

All AEs will be followed until they have abated, or until a stable situation has been reached. Depending on the event, follow up may require additional tests or medical procedures as indicated, and/or referral to the general physician or a medical specialist.
SAEs need to be reported till end of study within the Netherlands, as defined in the protocol

7.4 Safety Committee/DSMB

A DSMB is not deemed appropriate, considering that this study:

- Is not a high risk study, this is a moderate risk study
- Is not a study comparing treatments that are potentially lifesaving or preventing severe disease
- Does not contain an interim analysis
- Does not include patients that are especially vulnerable

8. STATISTICAL ANALYSIS

Patients who are included in the study and decide to withdrawal before start of treatment will be replaced by another participant. Patients who smoke during the study will be excluded from analysis. Patients who withdrawal early if the treatment already started will be evaluated. Analyses will be performed with the intention-to-treat population.

8.1 Primary study parameter(s)

The primary study parameter will be presented in percentage. Missing data is not applicable for this parameter. We consider the treatment on MR-Linac feasible if for at least in 80% of the patients (16/20 patients) 5 out of 5 fractions could be performed on MR-Linac. A fraction is technical feasible if:

- Treatment time is <1 hour. The (ATS) workflow includes: MR-scans for position verification, evaluation and adaptation of the contours by a radiation oncologist, re-optimizing the treatment plan and radiation dose delivery.
- The doses on the OARs deviates less than 5% from the pre-treatment plan doses.
- The maximum dose on the PTV deviates less than 5% from the pre-treatment plan dose and does not exceed a maximum of 120%.
- Patients can endure the treatment

Technical problems at the scanner, such as downtime of the machine, will not be considered as feasibility constraints.

8.2 Secondary study parameter(s)

Normally distributed continuous variables will be presented as mean with standard deviation. Non-normally distributed continuous variables will be presented as median with inter-quartile range. As the number of patients is low, non-parametric normally distributed statistical analysis will be performed on secondary outcome measures (QLQ, RT toxicity and voice quality) to compare baseline scoring with time points at the end of radiotherapy treatment. Time-to-event data (regional recurrence will be analysed with the Kaplan-Meier method. Patient reported outcomes (quality of life and treatment tolerability) will be analyzed with a mixed model.

8.3 Other study parameters

Descriptive statistics will be used for baseline patient, treatment and tumour characteristics.

9. ETHICAL CONSIDERATIONS

9.1 Regulation statement

The study will be conducted according to the principles of the Declaration of Helsinki (Helsinki, 2024) and in accordance with the Medical Research Involving Human Subjects Act (WMO) and this protocol. The protocol is submitted to the Independent Ethics Committee (IEC) and to the Radiation Protection Committee of the UMC Utrecht. The study will not start until the investigator has received written approval of the METC NedMec, and approval of the local board of the center.

9.2 Recruitment and consent

Patients with cT1-2 glottic cancer will be discussed in the multidisciplinary head and neck oncology meeting. Eligible patients will be notified about the ARIA study by their treating radiation oncologist. Patients who are interested, will be called by the responsible researcher to explain the study procedures. Patients willing to participate will sign the informed consent of the ARIA study together with the responsible researcher. The minimal time between informing the patient and signing the informed consent form is three days. The patient information letter and informed consent form is attached as a separate document

9.3 Benefits and risks assessment, group relatedness

Patients in the ARIA study will receive 5 fractions instead of 25 fractions. This will reduce the patient and hospital burden. The treatment time on MR-Linac will be longer compared to the conventional treatment (1 hour vs 15 minutes). If patients cannot endure the treatment on the MR-linac it is still possible to finish the treatment on the conventional linac. Switching to conventional treatment will not have consequence for the treatment outcome.

High doses on the larynx could result in more radiation toxicity. However, the pre-treatment dose constraints are based on the study of Sher et al., where radiation toxicity outcomes were promising (0% grade 3 or higher late toxicity). Additionally, we only include non-smoking patients. Since smoking is expected to be one of the largest risk factors for developing radiation toxicity, we expect that we have minimized the risks of high grade radiation toxicity.

Hearing protection (ear plugs) is provided in compliance with standard procedures, and hearing loss is therefore not expected to occur as a result of the noise exposure caused by the MR. Thereby, the treatment consists of only 5 fractions, which will minimize this risk.

9.4 Compensation for injury

The sponsor/investigator has a liability insurance which is in accordance with article 7 of the WMO. The sponsor (also) has an insurance which is in accordance with the legal requirements in the Netherlands (Article 7 WMO). This insurance provides cover for damage to research subjects through injury or death caused by the study. The insurance applies to the damage that becomes apparent during the study or within 4 years after the end of the study.

9.5 Incentives and reimbursements

Patients participating in the study are not eligible to receive travelling reimbursement, since there are no extra study visits to the UMC Utrecht. No further incentives or reimbursements are applicable.

10. ADMINISTRATIVE ASPECTS, MONITORING AND PUBLICATION

10.1 Handling and storage of data and documents

Two databases (clinical and technical) will be developed to collect all data. Patient, tumour and outcome characteristics including recurrences and CTCAE will be collected from HiX and registered in the clinical ARIA database. An eCRF will be created for the clinical ARIA database. The CRF will be stored in CASTOR (https://www.castoredc.com/) on a local server in the UMC-Utrecht. The technical database will be filled with specifications of RT treatment and imaging before, during and after treatment. Images are stored in the radiotherapy clinical image database as patients are treated in a clinical setting. Table 3 provides an overview of the complete data collection.

Central data management will be organized by the local trial supporting staff. The research data will be handled confidentially and encoded. After the informed consent is signed, each patient receives an unique study number. These codes will not be based on the patient initials and birth date. A subject identification code list will be used to link the data to the subject. The key to the code is safeguarded by the principle investigator and the datamanager. Data will be kept for 15 years. The subjects privacy is protected by the usage of the study number, instead of personal data. The handling of personal data will comply with the Dutch Personal Data Protection Act (De Wet Bescherming Persoonsgegevens). For further details regarding handling and storage of personal data, see the attached data management plan.

ARIA clinical database	Pre-treatment	During treatment	End of treatment	3m FU	6m FU	9m FU	12m FU	18m FU	24m FU
**Baseline**									
1. Enrollment	x								
2. Patient characteristics	x								
3. Tumour characteristics	x								
**Treatment**									
4. Radiotherapy dose prescription	x								
5. Radiotherapy delivered dose			x						
**Outcome**									

6. Survival and recurrence				x	x	x	x	x	x
7. Toxicity		x	x	x	x	x	x	x	x
8. Patient reported outcomes	x	x		x	x		x		x
9. Voice Handicap Index	x		x	x	x		x		x
10. (Serious) Adverse events	x	x	x	x	x	x	x	x	x

**ARIA technical database**	**Pre-treatment**	**During treatment**	**End of treatment**	**3m FU**	**6 m FU**	**9 m FU**	**12 m FU**	**18 m FU**	**24 m FU**
a. Imaging	X	X	X	X					
b. Radiotherapy specifics (RT struct, RT dose)			X						

1) Check inclusion criteria, check absence of exclusion criteria, date of signing informed consent, additional comments

2) Age at inclusion, gender, smoking status (if yes peak years, stop date), drinking status (if yes units per week), pain medication (if yes, which and amount) , additional comments

3) Date of diagnosis, date of MRI on baseline, date of CT on baseline, clinical tumour stage at diagnosis, additional comments

4) Date of first RT fraction

5) Date of last RT fraction, MRL treatment completed as planned? (if no: What was the reason for delay or early stop of RT?)

6) Local recurrence (yes/no), date of local recurrence, vital status: alive, left, deceased (if left or deceased: date of leaving study)

7) CTCAE v.5. Acute toxicity (Radiation dermatitis, Laryngeal/pharyngeal mucositis, dysphagia) and late toxicity (dysphagia, osteonecrosis, laryngeal edema and pharyngeal necrosis)

8) After 1st and 5th fractions: MRL-PRE questionnaire. At baseline and during FU: quality of life questionnaire (EORTC QLQ – C30, EORTC QLQ – HN35)

9) VHI: Voice Handicap Index questionnaires

10) As described under chapter 8

a) Planning CT/MRIs, MRI's during treatment, FU MRI at 3 months

b) All named structures and delineations, dose maps, re-planning events, dose volume histograms

10.2 Monitoring and Quality Assurance

External monitoring of this moderate risk trial will be performed by Julius Clinical (https://www.juliusclinical.com/) or any other independent CRO offering monitoring services. The trial management is organized by the researchers involved in the study. Since this is a monocenter study with a relative low number of patients, no external trial management agencies will be involved. All personnel has been trained for WMO-GCP or BROK®. The monitoring will be performed according to the NFU (Nederlandse Federatie van Universitaire Medische Centra) guidelines. The monitoring plan is attached as an addendum

10.3 Amendments

Amendments are changes made to the research after a favourable opinion by the accredited METC has been given. All amendments will be notified to the METC that gave a favourable opinion. Changes in the technical document will not be presented to the METC since this will not change the treatment given to the participants

10.4 Annual progress report

The sponsor/investigator will submit a summary of the progress of the trial to the accredited METC once a year. Information will be provided on the date of inclusion of the first subject, numbers of subjects included and numbers of subjects that have completed the trial, serious adverse events/ serious adverse reactions, other problems, and amendments.

10.5 Temporary halt and (prematurely) end of study report

The investigator/sponsor will notify the accredited METC of the end of the study within a period of 8 weeks. The end of the study is defined as the last patient's last visit.
The sponsor will notify the METC immediately of a temporary halt of the study, including the reason of such an action.
In case the study is ended prematurely, the sponsor will notify the accredited METC within 15 days, including the reasons for the premature termination.
Within one year after the end of the study, the investigator/sponsor will submit a final study report with the results of the study, including any publications/abstracts of the study, to the accredited METC.

10.6 Public disclosure and publication policy

After finishing the study, all results of this study will be publicized in an international journal which focuses on the treatment head and neck cancer. Publications related to this study will

be coordinated by the leading and assistant investigator. Investigators will all become co-author, according to the effort they put in a certain article related to this study.

11. STRUCTURED RISK ANALYSIS

11.1 Potential issues of concern

The clinical trial of Sher et al. (4) reported 1 local recurrence out of 21 patients who were treated with the 5x8.5 Gy fractionation scheme (4,8%). The study demonstrated excellent short-and long-term tolerability, with no patients experiencing late soft tissue necrosis. Additionally, the VHI scores significantly improved over time in this study.

Comparing to our current fractionation scheme for these cT1-2 glottic larynx patients (25x2.4 Gy) treated at UMC Utrecht (1), our current local recurrence was 7/93 patients (7.53%) at UMC Utrecht. This is higher compared to the phase II clinical trial of Sher et al. The toxicity outcomes of the conventional treatment show a total number of 4 out of 93 (4.3%) patients that experienced a grade 3 or higher late toxicity, compared to 0% late toxicities of this grade in the clinical trial of Sher et al. While we acknowledge that this clinical trial is still small, the promising outcomes suggest that his approach holds considerable potential.

11.2 Synthesis

To reduce the risk of more radiation toxicities compared to the conventional treatment, we only include non-smoking patients. Thereby, dosimetric constraints are set to ensure dose reduction on the organs at risk, we do not include patients who cannot meet these constraints. We believe that the benefits of reducing the treatment regimen from 25 to 5 fractions outweigh the possible risks of altered local recurrence and toxicity.

12. REFERENCES

1. de Ridder M, Rijken JA, Smits HJG, Smid EJ, Doornaert PAH, de Bree R. Oncological outcome of vocal cord-only radiotherapy for cT1-T2 glottic laryngeal squamous cell carcinoma. European Archives of Oto-Rhino-Laryngology. 2023;280(7):3345-52.
2. Tans L, Al-Mamgani A, Kwa SLS, Elbers JBW, Keskin-Cambay F, Sewnaik A, et al. Single vocal cord irradiation for early-stage glottic cancer: Excellent local control and favorable toxicity profile. Oral Oncology. 2022;127:105782.
3. Kang B-H, Yu T, Kim JH, Park JM, Kim J-I, Chung E-J, et al. Early Closure of a Phase 1 Clinical Trial for SABR in Early-Stage Glottic Cancer. International Journal of Radiation Oncology*Biology*Physics. 2019;105(1):104-9.
4. Sher DJ, Avkshtol V, Moon D, Vo D, Mau T, Childs L, et al. Stereotactic ablative radiotherapy for T1-2 glottic larynx cancer: mature results from the phase II LT-SABR trial. International Journal of Radiation Oncology, Biology, Physics.
5. Barnes H, Alexander S, Bower L, Ehlers J, Gani C, Herbert T, et al. Development and results of a patient-reported treatment experience questionnaire on a 1.5 T MR-Linac. Clinical and translational radiation oncology. 2021;30:31-7.
6. de Mol van Otterloo SR, Westerhoff JM, Leer T, Rutgers RHA, Meijers LTC, Daamen LA, et al. Patient expectation and experience of MR-guided radiotherapy using a 1.5T MR-Linac. Technical Innovations & Patient Support in Radiation Oncology. 2024;29:100224.
