# Supplementary material for: MR-guided ultra-hypofractionated radiotherapy for early-stage glottic cancer: A phase I feasibility study
Source: Clin Transl Radiat Oncol. 2026 Sep 22;62:101297. doi: 10.1016/j.ctro.2026.101297 (PMC13635521; doi:10.1016/j.ctro.2026.101297)
Supplement: Supplementary file 2 [file mmc2.pdf]

Supplementary file 2: scan parameters

The following scans are acquired on the 3 T MR scanner. Set-up is done with a single Flex S coil (Figure S1). These scans are used for pre-treatment delineation and radiotherapy planning.

**Parameters**	T2-weighted Turbo Spin Echo (TSE) – high resolution	T2-weighted Turbo Spin Echo (TSE)*	T1-weighted Turbo Spin Echo (TSE)	T1-weighted Turbo Spin Echo (TSE) with gadolinium
Field of View (RL x AP x FH) [mm]	250 x 194 x 50	550 x 251 x 120	250 x 198 x 50	250 x 198 x 50
Number of slices	25	60	25	25
Matrix size	416 x 324	552 x 248	556 x 440	416 x 329
Reconstructed resolution [mm^2]	0.43 x 0.43	0.49 x 0.49	0.22 x 0.22	0.43 x 0.43
Slice thickness [mm]	2	2	2	2
Slice gap [mm]	0	0	0	0
Compressed sense (CS)	1.4	2	2	2
TR [ms]	2818	4335	610	728
TE [ms]	100	80	9.3	14
Refocusing angle [degrees]	100	100	100	100

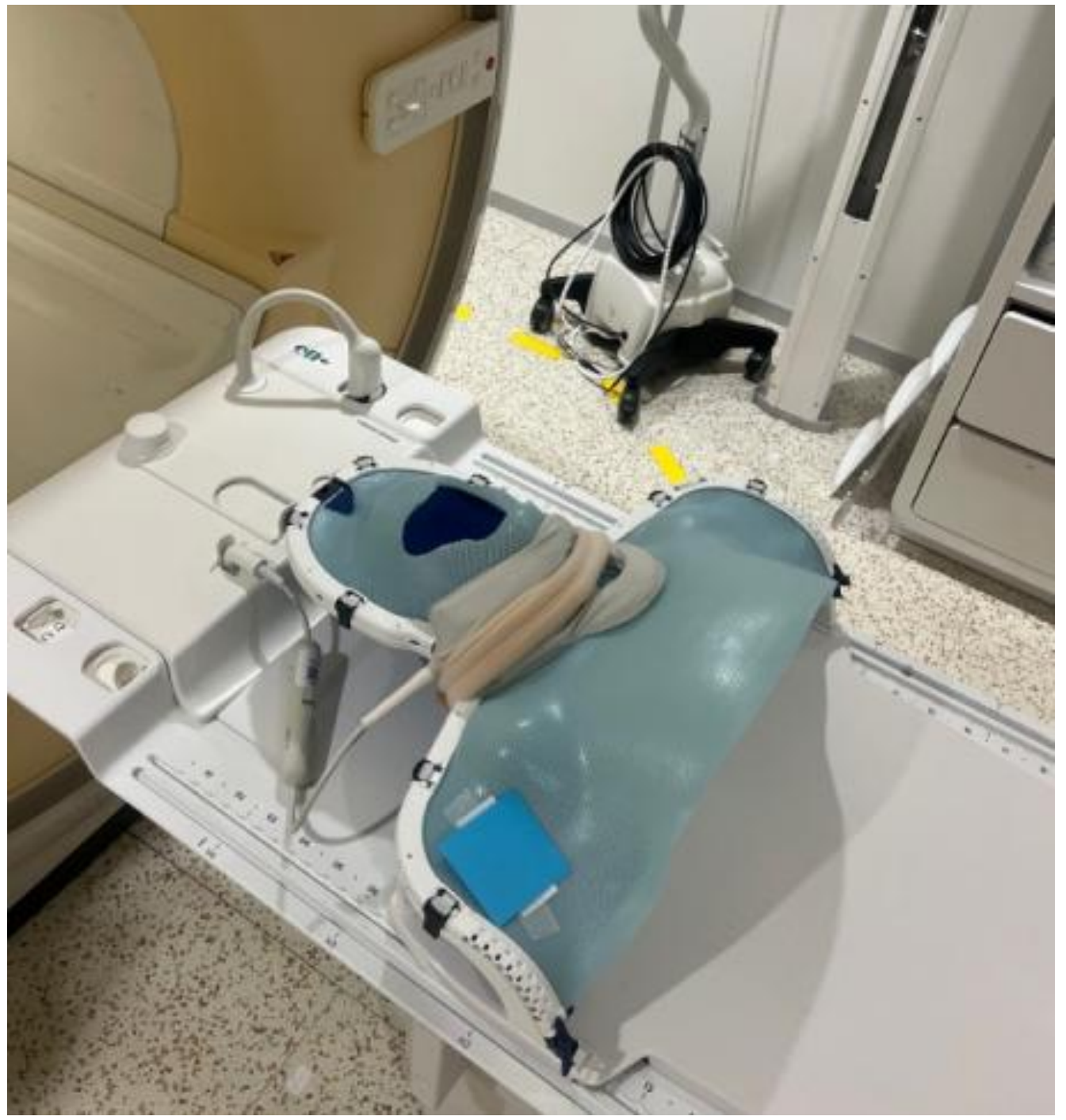

Figure S1 Set-up for pre-treatment scanning on 3T MR scanner

Number of averages (NSA)	2	2	2	2
Fat suppression	mDixon	-	-	mDixon

*This scan is used as a master for delineation of the tumor and organs at risk.

The following scan is acquired on the 1.5 T MR-Linac. This scan is used for daily adaptation of the delineations.

**Parameters**	T2-weighted multislice Turbo Spin Echo (TSE)
Field of View (RL x AP x FH) [mm]	550 x 248 x 120
Number of slices	59
Matrix size	460 x 196
Reconstructed resolution [mm^2]	0.86 x 0.86
Slice thickness [mm]	4
Slice gap [mm]	-2
Compressed sense (CS)	2
TR [ms]	4793
TE [ms]	80
Refocusing angle [degrees]	100
Number of averages (NSA)	2
Fat suppression	-
