# Supplementary material for: MR-guided ultra-hypofractionated radiotherapy for early-stage glottic cancer: A phase I feasibility study
Source: Clin Transl Radiat Oncol. 2026 Sep 22;62:101297. doi: 10.1016/j.ctro.2026.101297 (PMC13635521; doi:10.1016/j.ctro.2026.101297)
Supplement: Supplementary file 3 [file mmc3.pdf]

Supplementary file 3: Margins gating envelope

**Tumor side**	**Right**	**Left**	**Bilateral**
	Margin to GTV (mm)	Margin to GTV (mm)	Margin to GTV (mm)
Left	2	3	2
Right	3	2	2
Anterior	2	2	2
Posterior	3	3	3
Caudal	4	4	4
Cranial	4	4	4
